# Vemurafenib in Chinese patients with *BRAF*^V600^ mutation–positive unresectable or metastatic melanoma: an open-label, multicenter phase I study

**DOI:** 10.1186/s12885-018-4336-3

**Published:** 2018-05-03

**Authors:** Lu Si, Xiaoshi Zhang, Zhen Xu, Qiudi Jiang, Lilian Bu, Xuan Wang, Lili Mao, Weijiang Zhang, Nicole Richie, Jun Guo

**Affiliations:** 1 Key Laboratory of Carcinogenesis and Translational Research (Ministry of Education), Peking University Cancer Hospital & Institute, 52# Fucheng Road, Haidian District, Beijing, 100142 China; 2 Sun Yat-sen University Cancer Center, 651 Dongfeng R. E, Guangzhou, 510060 China; 3 Roche Product Development in Asia Pacific, 1100 Longdong Ave, Pudong District, Shanghai, 201203 China; 4 Roche R&D Center China Ltd., 917 Ha Lei Road, Building 6, Pudong District, Shanghai, 201203 China; 5 Roche Innovation Center New York, 430 E. 29th Street, New York, NY 10016 USA; 6 Genentech, Inc., 1000 National Ave 231, San Bruno, California, 94066 USA

**Keywords:** *BRAF*^V600^-positive, Advanced melanoma, Vemurafenib, China, Asia

## Abstract

**Background:**

Melanoma is a rare, deadly disease without effective treatment options in China. Vemurafenib is a selective inhibitor of oncogenic *BRAF*^V600^ kinase approved in more than 90 countries, based on results obtained primarily in Caucasian patients. Limited data are available regarding the efficacy and safety of vemurafenib in Asian patients.

**Methods:**

This phase I study investigated the pharmacokinetics, efficacy, and tolerability of vemurafenib (960 mg twice daily) in Chinese patients with *BRAF*^V600^ mutation–positive unresectable or metastatic melanoma. The study included two cohorts: a pharmacokinetic cohort (*n* = 20) and an expansion cohort (*n* = 26).

**Results:**

After 21 days of dosing, vemurafenib demonstrated marked accumulation and relatively constant steady-state exposure over the dosing period. Confirmed best overall response rate was 52.2% (95% CI 37.0–67.1%). Median progression-free survival was 8.3 months (95% CI 5.7–10.9%); median overall survival was 13.5 months (95% CI 12.2%–not estimable). The most common adverse events were dermatitis acneiform, arthralgia, diarrhea, blood cholesterol level increase, blood bilirubin level increase, melanocytic nevus, and alopecia. A total of nine grade 3 or 4 adverse events were reported in seven patients (15.2%).

**Conclusion:**

Overall, vemurafenib showed a favorable benefit-risk profile among Chinese patients. Pharmacokinetics, safety, and efficacy were generally consistent with those reported in Caucasian patients.

**Trial registration:**

ClinicalTrials.gov identification: NCT01910181. Registered 29 July 2013, prospectively registered.

**Electronic supplementary material:**

The online version of this article (10.1186/s12885-018-4336-3) contains supplementary material, which is available to authorized users.

## Background

Melanoma is relatively rare in China, with a reported incidence of 0.6 per 100,000 persons in 2012 [1]. However, most Chinese patients with melanoma (> 90%) are diagnosed with locally advanced disease (stage II or higher) and prognosis is poor, particularly for those with stage IV disease, among whom the estimated 5-year survival rate is 4.6% [2].

Several new treatment options for metastatic melanoma have emerged in the past 5 years, including monoclonal antibodies targeting the programmed cell death 1 receptor and cytotoxic T-lymphocyte–associated antigen 4 and small-molecule inhibitors of BRAF and MEK. However, clinical trials for these agents have been conducted almost exclusively in Caucasian populations. Consequently, these agents are largely considered investigational in China [3]. Despite safety concerns and poor overall survival (OS), dacarbazine has remained standard first-line therapy for metastatic melanoma in China [3, 4].

Approximately 50% of melanomas in Caucasian patients harbor *BRAF* mutations, resulting in constitutive *BRAF* kinase activity [5–7]. Among Chinese patients with melanoma, the rate of *BRAF* mutation is approximately 25% [8]. Vemurafenib is a highly selective inhibitor of oncogenic *BRAF* kinase. In the pivotal phase III study (BRIM-3), vemurafenib significantly improved progression-free survival (PFS) and OS compared with dacarbazine in patients with *BRAF*^V600^-mutated metastatic melanoma [9, 10]. More than 99% of patients enrolled in BRIM-3 were Caucasian [9], and limited data are available regarding the efficacy and safety of vemurafenib in Asian patients. This phase I study evaluated the pharmacokinetics, efficacy, and safety of vemurafenib in Chinese patients with *BRAF*^V600^ mutation–positive unresectable or metastatic melanoma.

## Methods

### Study design

This open-label, multicenter, phase I study enrolled two cohorts: a pharmacokinetic cohort and an expansion cohort. For the pharmacokinetic cohort, patients received treatment in three periods after a 28-day screening period. In period A (days 1–21), patients received oral vemurafenib 960 mg twice daily. Patients fasted overnight for at least 8 h before and at least 4 h after the morning dose on days 1 and 21. Light snacks (crackers, toast, water, and juice) were allowed in the 4-h post-dose period. All other doses were administered either 1 h before or 2 h after a meal. Only the morning dose was administered on day 21. In period B (days 22–27), patients temporarily discontinued vemurafenib to enable characterization of the elimination profile. In period C (day 28 onwards), patients continued to receive vemurafenib 960 mg twice daily until disease progression, unacceptable toxicity, withdrawal of consent, or discontinuation for any other reason.

Enrollment in the expansion cohort was initiated after completion of recruitment into the pharmacokinetic cohort. In the expansion cohort, all patients received vemurafenib 960 mg twice daily until progressive disease, unacceptable toxicity, withdrawal of consent, or discontinuation for any other reason. Dose modifications (temporary interruption or dose reductions) were allowed within both cohorts for management of symptomatic adverse events (AEs).

This study was conducted in accordance with International Congress on Harmonisation guidelines for Good Clinical Practice, the principles of the Declaration of Helsinki, and local regulations. All patients provided written informed consent. This trial was registered prospectively at ClinicalTrials.gov (ID, NCT01910181) on July 29, 2013. The trial was designed jointly by the corresponding author and the sponsor (F. Hoffmann-La Roche Ltd). Data were collected by the site investigators and were retained and analyzed by the sponsor. All authors had full access to the data.

### Patients

Chinese patients ≥18 years of age with histologically confirmed metastatic melanoma (unresectable stage IIIC or stage IV) positive for *BRAF*^V600^ mutation by cobas® BRAF V600 Mutation Test (Roche Molecular Diagnostics, Pleasanton, CA, USA) were eligible. Other key inclusion criteria included measurable disease according to Response Evaluation Criteria in Solid Tumors, version 1.1 (RECIST v1.1); Eastern Cooperative Oncology Group performance status 0–1; adequate hematologic, renal, and liver function; life expectancy > 3 months; and absence of active central nervous system metastases. Patients could be treatment-naive or have previously received systemic therapy (excluding BRAF or MEK inhibitors).

### Outcomes and assessments

The primary objective was to evaluate the pharmacokinetics of vemurafenib 960 mg twice daily in Chinese patients, including maximal concentration (C_max_); time to maximal concentration (t_max_); area under the concentration-time curve from 0 to 8 hours (AUC_0-8h_)_,_ AUC_0-12h,_ and AUC_0-168h;_ trough concentration (C_trough_); accumulation ratio (defined as AUC_0-8h_ on day 21/AUC_0-8h_ on day 1); terminal elimination rate constant (K_el_); and terminal half-life (t_½_). The schedule of assessments is in the Additional file 1. Pharmacokinetic parameters were calculated using noncompartmental analysis (Phoenix WinNonlin, version 6.2; Pharsight, a Certara company, Princeton, NJ, USA).

Efficacy endpoints included best overall response rate (BORR) according to RECIST v1.1, confirmed by repeat assessment at least 4 weeks after criteria were first met, PFS, OS, and duration of response (DOR). Tumor assessments (computed tomography or magnetic resonance imaging) were obtained at screening, day 1 of cycle 3, and every 2 cycles thereafter (or as clinically indicated) until documented disease progression. DOR, PFS, and OS were estimated using the Kaplan-Meier method.

Safety assessments consisted of monitoring and recording of AEs, serious AEs, and nonserious AEs of special interest; protocol-specified laboratory assessments; vital signs; and other protocol-specified tests. AEs were graded according to National Cancer Institute Common Terminology Criteria for Adverse Events, version 4.0. Patients were followed for safety for 28 days after the last dose of study medication or until resolution of drug-related AEs. All patients who received at least one dose of vemurafenib were to be followed for evaluation of cutaneous squamous cell carcinoma (cuSCC) until 6 months after discontinuation of study treatment, or until death, withdrawal of consent, or loss to follow-up, whichever occurred first.

The pharmacokinetic analysis population included all patients who provided sufficient pharmacokinetic data to obtain at least one of the primary pharmacokinetic variables. The safety population included all patients who received at least one dose of vemurafenib, and the tumour response population included all patients.

### Statistical analysis

No formal sample size calculation was performed. The sample size of 20 patients in the pharmacokinetic cohort was chosen to characterize the vemurafenib pharmacokinetic profile with consideration of interpatient variability. With 20 patients enrolled in the pharmacokinetic cohort, at least 10 patients would be expected to complete the pharmacokinetic portion of the study. A total of 45 patients (20 in the pharmacokinetic cohort and 25 in the expansion cohort) would give a Clopper-Pearson 95% CI of 26–56% for BORR, assuming the target BORR (confirmed) is 40%.

## Results

### Patients

Forty-six patients were enrolled between August 17, 2013, and January 24, 2014 at two centers in China. Twenty patients were enrolled in the pharmacokinetic cohort and 26 were enrolled in the expansion cohort. Baseline demographics and disease characteristics are shown in Table 1. At the time of data cut-off (December 15, 2014), 14 patients (30%) remained on treatment and 32 (70%) had discontinued study treatment because of disease progression (*n* = 28), patient withdrawal (*n* = 2), or other reasons (*n* = 2). The median follow-up duration was 11.3 months (range 3.3–16.0).

**Table 1:** Baseline characteristics

	Vemurafenib (*N* = 46)
Age, y, median (range)	42 (19–69)
Male, n (%)	21 (46)
ECOG performance status, n (%)	
0	30 (65)
1	16 (35)
Serum LDH level, n (%)	
Normal	27 (59)
Elevated	19 (41)
Histologic subtype, n (%)	
Acral lentiginous	1 (2)
Nodular	7 (15)
Pigmented nevus	1 (2)
Superficial spreading	14 (30)
Unknown	23 (50)
Median time since diagnosis of metastatic disease (range), months	4.5 (0–31.0)
Disease stage, n (%)	
Unresectable stage IIIC	3 (7)
Stage IV	43 (94)
M1a^a^	9 (21)
M1b^a^	8 (19)
M1c^a^	26 (61)
Prior treatment for metastatic disease, n (%)	31 (67)
Number of prior therapies for metastatic disease, n (%)	
0	15 (33)
1	21 (46)
2	6 (13.0)
≥ 3	3 (7)
Unknown	1 (2)
Previous CTLA-4 or PD-1/PD-L1 inhibitor, n (%)	0 (0)

*ECOG* Eastern Cooperative Oncology Group, *LDH* lactate dehydrogenase; *CTLA-4* cytotoxic T-lymphocyte-associated protein 4; *PD-1* programmed death 1; *PD-L1* programmed death ligand 1

^a^Denominator is the number of patients with stage IV disease (*n* = 43)

### Pharmacokinetics

All patients in the pharmacokinetic cohort were included in the pharmacokinetic analysis population; one patient had treatment interruption on day 14 of cycle 1 and was excluded from pharmacokinetic analysis for subsequent time points. Additionally, one patient was excluded from analysis of t_½_ and volume of distribution because K_el_ could not be reliably estimated owing to insufficient concentration timepoint data during the elimination phase.

On day 1 after the first dose of vemurafenib 960 mg, mean vemurafenib concentrations reached C_max_ at approximately 5 h (median t_max_; range 2.0–8.0). Interpatient variability in AUC and C_max_ was relatively high, and was more pronounced after the first dose than after multiple doses (Table 2). On day 21, mean plasma concentrations of vemurafenib remained relatively constant throughout the 24-h period after the morning dose. Median t_max_ was 1 h (range 0.0–5.0) after the morning dose on day 21. During the 168-h drug holiday after the morning dose on day 21, vemurafenib plasma concentrations decreased, with a mean terminal t_½_ of approximately 35.6 h. After 21 days of vemurafenib 960 mg twice-daily dosing, use of vemurafenib resulted in extensive accumulation (≈18-fold) and relatively constant steady-state exposure throughout the dosing interval (Table 2). In the pharmacokinetic cohort, mean trough concentrations continually increased from days 15 to 21 (Table 2). Mean vemurafenib trough concentrations in both the pharmacokinetic and expansion cohorts seemed lower in later cycles than in earlier cycles (Additional file 2: Figure S1).

**Table 2:** Vemurafenib pharmacokinetics in the pharmacokinetic cohort

		Vemurafenib (*N* = 20)	
Study day	Parameter	Mean ± SD	CV %
Day 1	AUC_0-8h_, μg·h/mL	37.5 ± 22.3	59.4
	AUC_0-12h_, μg·h/mL	57.5 ± 32.6	56.7
	C_max_, μg/mL	6.9 ± 3.9	55.8
Day 15	C_trough_, μg/mL	63.0 ± 23.3	37.0
Day 21	AUC_0-8h_, μg·h/mL	501.3 ± 123.0	24.5
	AUC_0-12h_, μg·h/mL	720.3 ± 185.8	25.8
	C_max_, μg/mL	77.6 ± 17.9	23.0
	C_trough_, μg/mL	72.6 ± 20.0	27.5
	t_½_, h	35.6 ± 18.1	–
	Accumulation ratio	17.9 ± 14.1	–

*SD* standard deviation, *CV* coefficient of variation, *AUC*_*0-8h*_ area under the concentration-time curve from 0 to 8 h, *AUC*_*0-12h*_ area under the concentration-time curve from 0 to 12 h, *C*_*max*_ maximal plasma concentration, *C*_*trough*_ plasma trough concentration, *t*_*½*_ terminal half-life, *accumulation ratio* AUC_0-8h_ on day 21/AUC_0-8h_ on day 1

### Efficacy

**Fig. 1:**
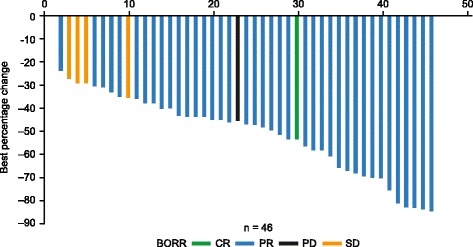
Best percentage change from baseline in target lesion size in individual patients. The patient with CR had only 1 target lesion: a lymph node that shrunk to < 10 mm. BORR = best overall response rate; CR = complete response; PR = partial response; PD = progressive disease; SD = stable disease

All patients (*n* = 46) were included in the efficacy analysis population. At clinical cut-off, one patient (2%) had confirmed complete response and 23 patients (50%) had confirmed partial response (Fig. 1). Median time to response was 1.8 months (range 1.7–5.7) by univariate analysis, and Kaplan-Meier–estimated median duration of response was 9.1 months (95% CI 7.4–not estimable [NE]) (Table 3). The confirmed BORR was 52% (95% CI 37–67%). An additional 21 patients (46%) had best response of stable disease, for an overall disease control rate (complete + partial response + stable disease) of 98% (95% CI 89–100%) (Table 3). A waterfall plot of best percentage change from baseline in target lesion size for each patient is shown in Fig. 1.

**Table 3:** Efficacy outcomes

Outcome	Vemurafenib (*N* = 46)
Confirmed BORR, n (%) [95% CI]	24 (52.2) [36.95–67.11]
Complete response	1 (2.2)
Partial response	23 (50.0)
Stable disease	21 (45.7)
Progressive disease	1 (2.2)
Disease control rate	45 (97.8)
Median time to confirmed response, months (range)	1.84 (1.7–5.7)
Median duration of confirmed response, months (95% CI)	9.13 (7.39–NE)
Median PFS, months (95% CI)	8.25 (5.65–10.94)
PFS, % (95% CI)	
6 months	58.7 (44.5–72.9)
1 year	33.4 (19.5–47.4)
Median OS, months (95% CI)	13.54 (12.19–NE)
OS, % (95% CI)	
6 months	89.1 (80.1–98.1)
1 year	64.6 (50.7–78.6)

*BORR* best overall response rate, *CI* confidence interval, *NE* not estimable, *PFS* progression-free survival, *OS* overall survival

At the clinical cut-off date, 32 patients (70%) had experienced a PFS event (disease progression or death). Kaplan-Meier–estimated median PFS was 8.3 months (95% CI 5.7–10.9) (Table 3; Additional file 3: Figure S2). Twenty patients (44%) had experienced an OS event (death). Kaplan-Meier–estimated median OS was 13.5 months (95% CI 12.2–NE) (Table 3; Additional file 3: Figure S2).

### Safety

All patients (*n* = 46) received at least one dose of study medication and were included in the safety analysis. The median duration of treatment was 7.9 months (range 1.8–16.0).

All patients experienced at least one AE; the most common AEs (occurring in ≥20% patients) are reported in Table 4. AEs leading to dose modification or interruption of vemurafenib were reported in 10 patients (22%); no AEs leading to treatment discontinuation were reported. A total of nine grade ≥ 3 AEs were reported in seven patients (15%), regardless of relationship to study drug. Grade ≥ 3 AEs included anemia in two patients and lymphopenia, chest discomfort, hypokalemia, blood alkaline phosphatase level increase, blood cholesterol level increase, uveitis, and γ-glutamyltransferase level increase in one patient each. Two patients (4%) had serious AEs within the reporting period (grade 3 chest discomfort and grade 3 uveitis), both of which were considered related to vemurafenib. No grade 5 AEs occurred during the reporting period, and no cases of cuSCC, keratoacanthoma, or Bowen’s disease were reported.

**Table 4:** Most common AEs occurring in ≥20% of patients

AE, n (%)	Vemurafenib (*N* = 46)	
	Any grade	Grade ≥ 3
At least 1 AE	46 (100)	7 (15.2)
Dermatitis acneiform	30 (65.2)	0
Arthralgia	30 (65.2)	0
Blood cholesterol level increase	27 (58.7)	1 (2.2)
Diarrhea	27 (58.7)	0
Blood bilirubin level increase	25 (54.3)	0
Melanocytic nevus	24 (52.2)	0
Alopecia	23 (50.0)	0
Palmar-plantar erythrodysesthesia syndrome	22 (47.8)	0
Photosensitivity reaction	17 (37.0)	0
Fatigue	14 (30.4)	0
Pyrexia	13 (28.3)	0
Rash maculopapular	12 (26.1)	0
γ-glutamyltransferase level increase	11 (23.9)	1 (2.2)
Proteinuria	11 (23.9)	0
Total bile acid level increase	10 (21.7)	0
Hypertriglyceridemia	10 (21.7)	0
Leukopenia	10 (21.7)	0

*AE* adverse event

## Discussion

To date, this is the largest study to evaluate the efficacy and safety of vemurafenib in an Asian population. Vemurafenib demonstrated marked accumulation and relatively constant steady-state exposure in Chinese patients, consistent with previous observations in predominantly Caucasian populations [11, 12]. Comparison of pharmacokinetic results in Chinese patients with melanoma in the current study with those in predominantly Caucasian patients in a previous study with a similar design show that vemurafenib exposures were higher in Chinese patients (Additional file 4: Table S1) [11]. On day 1, mean AUC_0-8h_ and C_max_ in Chinese patients were 39% and 44% higher, respectively, than those in predominantly Caucasian populations. More importantly, at steady state (day 15 in the previous Caucasian pharmacokinetic study and day 21 in the current study), mean AUC_0-8h_ and C_max_ (day 21) were 28% and 26% higher, respectively, than in predominantly Caucasian populations (day 15) [11].

These differences in exposure may be partially explained by increases in vemurafenib trough concentrations (C_trough_) from days 15 to 21, although it is unclear whether these increases in concentration were caused by continuous accumulation or intrapatient variability. While a lower average body weight is frequently identified as a possible reason for increased drug exposure in Asians relative to Caucasians, body weight or body mass index has not been identified as a covariant for vemurafenib pharmacokinetic parameters from population pharmacokinetic analysis of data from phase I, II, and III studies [13]. Furthermore, although exposure was higher in Chinese patients, it was within the range observed in predominantly Caucasian populations. Additionally, the differences in mean exposure are unlikely to lead to clinically meaningful differences in toxicity, given the previous demonstration of lack of a significant effect of vemurafenib exposure within the current range on liver laboratory values or skin toxicities [13]. Although exposure-dependent QT interval prolongation was observed in a substudy of the phase II BRIM-2 study [13], no patient in this study had a post-baseline corrected QT interval (Fridericia’s formula) > 500 msec.

The efficacy of vemurafenib in Chinese patients with *BRAF*^V600^-mutated unresectable or metastatic melanoma was similar to that observed in predominantly Caucasian populations (Additional file 5: Table S2) [10, 12]. In the current study, patients could have been treatment-naive or have previously received systemic therapy. Therefore, efficacy results were compared with patients treated with vemurafenib in the phase II BRIM-2 study (previously treated with at least one prior line of systemic therapy) [12] and the pivotal BRIM-3 study (treatment-naive) [10]. After similar median follow-up durations, the confirmed BORR in Chinese patients (52%) was similar to that reported in predominantly Caucasian populations with previously treated and treatment-naive disease (both 57%) [10, 12]. Although the CR rate in the current study in Chinese patients was numerically lower than that reported in the vemurafenib arm of BRIM-3 (2% vs. 6%, respectively), it remains comparable, as indicated by the overlapping 95% CI (0.06–11.53 in this study vs. 3.4–8.7 in BRIM-3) [10]. Furthermore, the percentage of LDH elevated and M1C was also comparable between the current study and BRIM-3 (LDH elevated: 41% vs. 42%; M1C: 61% vs. 66%, respectively). PFS (8.3 vs. 6.8 and 6.9 months, respectively) and OS (13.5 vs. 15.9 and 13.6 months, respectively) were also similar between Chinese and predominantly Caucasian populations [10, 12]. A post hoc subgroup analysis of the current study showed no substantial differences in efficacy (BORR, PFS, OS) among patients who were treatment-naive and those who had received one, two, or more than two previous lines of systemic therapy, although comparisons were limited by small patient numbers in each subgroup.

Vemurafenib was generally well tolerated in Chinese patients. Compared with predominantly Caucasian patients treated with vemurafenib in the pivotal BRIM-3 study [9, 10], Chinese patients had higher incidences of blood cholesterol level increase (59% vs. < 1%), hypertriglyceridemia (22% vs. < 1%), total bile acid increase (22% vs. 0%), hyperuricemia (17% vs. < 1%), blood bilirubin level increase (54% vs. 9%), leukopenia (22% vs. 0%), proteinuria (24% vs. < 1%), and melanocytic nevus (52% vs. 10%) (Additional file 6: Table S3) [10]. Lipid panels were not done as part of the BRIM-3 protocol, in contrast to formal data collection of the full fasting lipid profile required in the current study; as a result, no conclusions can be drawn from these comparisons. In the current study, the majority of these laboratory AEs met the criteria as AEs because the events were considered medically significant by investigators but were not accompanied by clinical symptoms, did not require a change in study treatment, and did not result in medical intervention. Moreover, apart from one event of grade 3 blood cholesterol level increase leading to treatment interruption, all of these events were grade 1–2, asymptomatic, and did not necessitate dose modification. Therefore, it is highly unlikely that these observed differences in clinical AEs impact the overall benefit/risk evaluation of vemurafenib.

In the BRIM-3 study, grade 3 cuSCC and grade 3 keratoacanthoma occurred in 19% (65 of 337) and 10% of patients (34 of 337) in the vemurafenib arm, respectively [9, 10]. In contrast, no such events were observed in Chinese patients in the current study. Similarly, no cuSCC or keratoacanthoma was observed in a phase I/II study of vemurafenib in Japanese patients with melanoma [14], and only one case of cuSCC was reported in an early post-marketing phase vigilance study that followed 95 vemurafenib-treated Japanese patients with metastatic melanoma [15]. This could potentially be related to differences between Asian and Caucasian populations. Because plasma concentrations of vemurafenib were generally consistent between Asian and Caucasian patients, this difference could be attributed to differences in culture and lifestyle, such as duration of exposure to sunlight, as well as differences in genetic susceptibility. Considering the relatively small sample sizes of the current study and the Japanese study (*N* = 11), actual incidences of these skin disorders in Asian patients must be confirmed in larger patient populations.

## Conclusions

In conclusion, vemurafenib pharmacokinetics, efficacy, and safety in Chinese patients were generally consistent with those observed in predominantly Caucasian populations, despite slightly higher vemurafenib exposure in Chinese patients. Overall, vemurafenib showed a favorable benefit/risk profile among Chinese patients.

## Additional files

Additional file 1: Schedule of assessments. (DOCX 19 kb)

Additional file 2: **Figure S1.** Vemurafenib C_trough_ concentrations (mean ± SD) after day 28 in the pharmacokinetics and expansion cohorts. *SD* standard deviation, *CV* coefficient of variation. (PDF 71 kb)

Additional file 3: **Figure S2.** Kaplan-Meier plots of (A) progression-free survival (PFS) and (B) overall survival (OS). (PDF 80 kb)

Additional file 4: **Table S1.** Comparison of pharmacokinetic parameters between study YO28390 (Chinese patients) and study NP25163 (predominantly Caucasian patients). (DOCX 19 kb)

Additional file 5: **Table S2.** Comparison of efficacy between study YO28390 (Chinese patients) and the BRIM-2 and BRIM-3 studies (predominantly Caucasian patients). (DOCX 18 kb)

Additional file 6: **Table S3.** Comparison of safety between study YO28390 (Chinese patients) and the BRIM-3 study (predominantly Caucasian patients). (DOCX 18 kb)

## Abbreviations

AEs: Adverse events
AUC: Area under the concentration-time curve; BORR: Best overall response rate
C_max_: Maximal concentration
C_trough_: Trough concentration
cuSCC: Cutaneous squamous cell carcinoma
DOR: Duration of response
K_el_: Terminal elimination rate constant
NE: Not estimable
OS: Overall survival
PFS: Progression-free survival
RECIST v1.1: Response Evaluation Criteria In Solid Tumors, version 1.1
t_1/2_: Terminal half-life
t_max_: Time to maximal concentration
