# Supplementary material for: Vemurafenib in Chinese patients with BRAFV600 mutation–positive unresectable or metastatic melanoma: an open-label, multicenter phase I study
Source: BMC Cancer. 2018 May 3;18:520. doi: 10.1186/s12885-018-4336-3 (PMC5934791; doi:10.1186/s12885-018-4336-3)
Supplement: Supplementary file 1 — Schedule of assessments. (DOCX 19 kb) [file 12885_2018_4336_MOESM1_ESM.docx]

**Additional file 1**

**METHODS**

***Schedule of Assessments***

PK parameters included C_max_ on days 1 and 21; t_max_ on days 1 and 21; AUC_0-8h_ and AUC_0-12h_ on days 1 and 21; AUC_0-168h_ beginning on day 21; C_trough_ on days 15, 19, and 21; accumulation ratio (defined as AUC_0-8h_ on day 21/AUC_0-8h_ on day1); K_el_; and t_½_. In the PK cohort, PK plasma samples were obtained pre-dose and at 1, 2, 4, 5, 8, and 12 hours post-dose on days 1 and 21, and up to 1 hour pre-dose on days 15 and 19 in period A. In period B, two PK samples were taken approximately 4 hours apart on day 22 (i.e., 24 hours and 28 hours after the morning dose on day 21) and on day 24 (i.e., 72 hours and 76 hours after the morning dose on day 21). One PK sample was taken up to 1 hour before the morning dose on day 28 in period C. Additionally, single PK samples were taken at the time of all tumor assessments, at the time of first disease progression, and as close as possible to the time of onset of any AE that led to dose adjustment or treatment interruption in all patients in the PK and expansion cohorts.
