# Supplementary figures and images for: Vemurafenib in Chinese patients with BRAFV600 mutation–positive unresectable or metastatic melanoma: an open-label, multicenter phase I study
Source: BMC Cancer. 2018 May 3;18:520. doi: 10.1186/s12885-018-4336-3 (PMC5934791; doi:10.1186/s12885-018-4336-3)

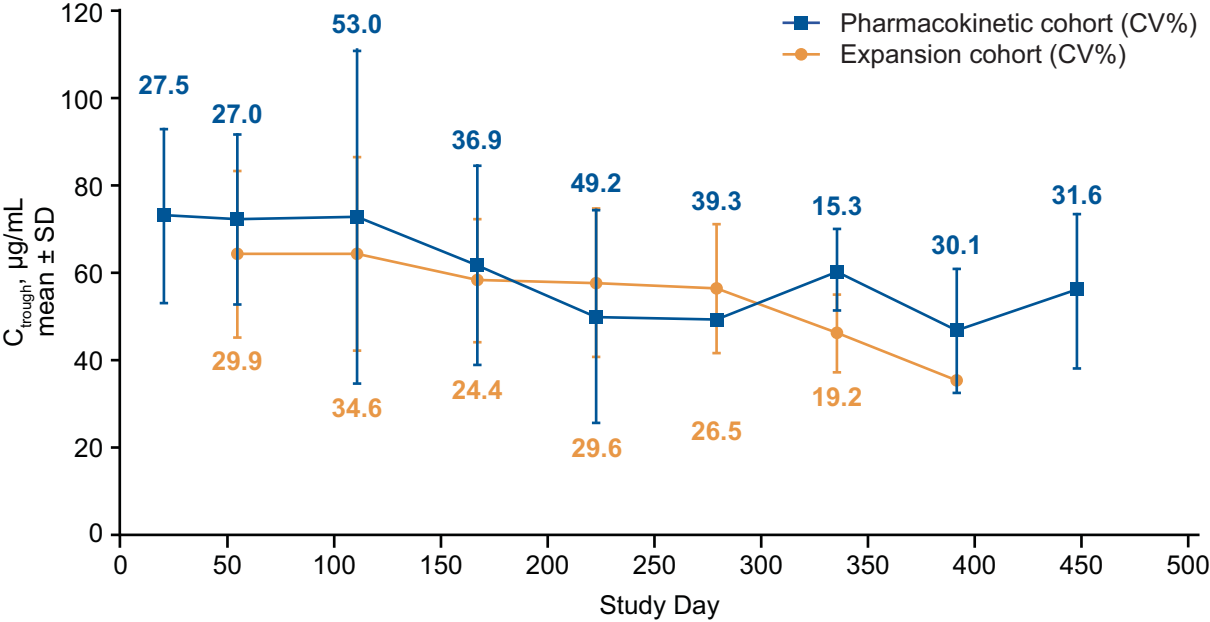

Supplement: Supplementary file 2 — Figure S1. Vemurafenib Ctrough concentrations (mean ± SD) after day 28 in the pharmacokinetics and expansion cohorts. SD standard deviation, CV coefficient of variation. (PDF 71 kb) [file 12885_2018_4336_MOESM2_ESM.pdf]

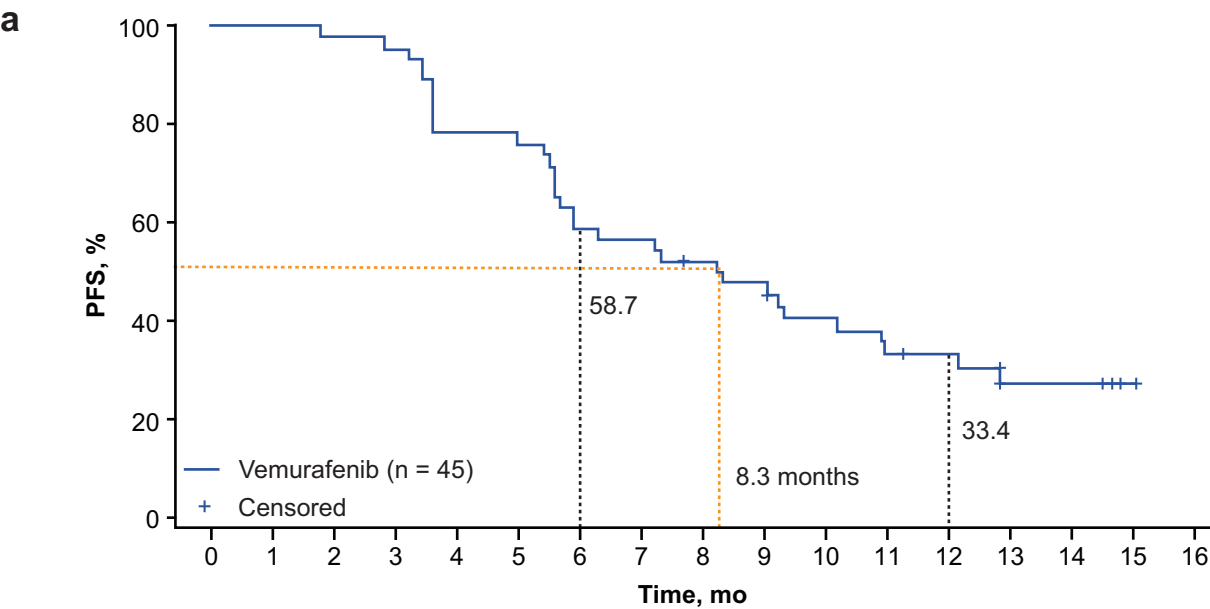

No. of patients at risk

Vemurafenib 46 46 45 44 36 35 27 26 23 21 17 14 12 6 6 1

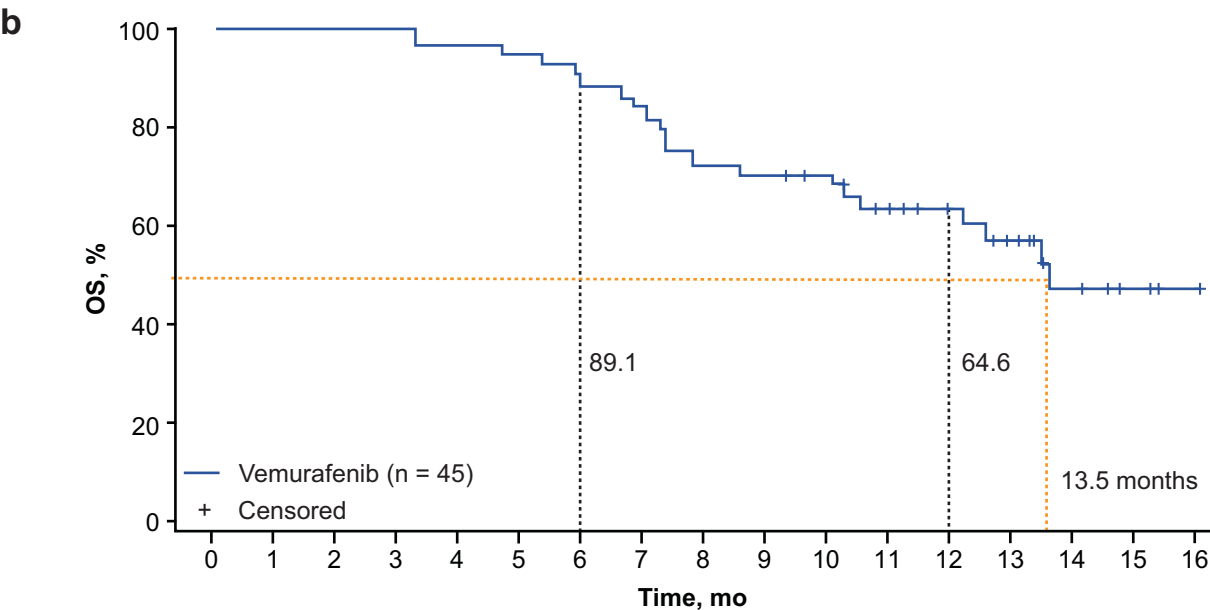

No. of patients at risk

Vemurafenib 45 46 46 46 45 44 41 38 34 33 31 25 21 17 9 4

Supplement: Supplementary file 3 — Figure S2. Kaplan-Meier plots of (A) progression-free survival (PFS) and (B) overall survival (OS). (PDF 80 kb) [file 12885_2018_4336_MOESM3_ESM.pdf]
