# Supplementary material for: Vemurafenib in Chinese patients with BRAFV600 mutation–positive unresectable or metastatic melanoma: an open-label, multicenter phase I study
Source: BMC Cancer. 2018 May 3;18:520. doi: 10.1186/s12885-018-4336-3 (PMC5934791; doi:10.1186/s12885-018-4336-3)
Supplement: Supplementary file 4 — Table S1. Comparison of pharmacokinetic parameters between study YO28390 (Chinese patients) and study NP25163 (predominantly Caucasian patients). (DOCX 19 kb) [file 12885_2018_4336_MOESM4_ESM.docx]

**Table S1** Comparison of PK parameters between Chinese and Caucasian patients

| Study day | PK parameters | Chinese patients  (study YO28390) | | Caucasian patients  (study NP25163) [11] | |
| --- | --- | --- | --- | --- | --- |
|  |  | Mean ± SD | CV % | Mean ± SD | CV % |
| Day 1 | AUC_0-8h_, µg·h/mL | 37.5 ± 22.3 | 59.4 | 27.0 ± 18.9 | 69.9 |
|  | C_max_, µg/mL | 6.9 ± 3.9 | 55.8 | 4.8 ± 3.3 | 69.8 |
| Day 15 | AUC_0-8h_, µg·h/mL | — | — | 392.2 ± 126.4 | 32.2 |
|  | C_max_, µg/mL | — | — | 61.4 ± 22.8 | 37.1 |
|  | C_trough_, µg/mL | 63.0 ± 23.3 | 37.0 | 55.4 ± 19.2 | 34.7 |
|  | t_½_, hours | — | — | 34.1 ± 19.7 | — |
|  | Accumulation ratio | — | — | 23.2 ± 16.5 | — |
| Day 21 | AUC_0-8h_, µg·h/mL | 501.3 ± 123.0 | 24.5 | — | — |
|  | C_max_, µg/mL | 77.6 ± 17.9 | 23.0 | — | — |
|  | C_trough_, µg/mL | 72.6 ± 20.0 | 27.5 | — | — |
|  | t_½_, hours | 35.6 ± 18.1 | — | — | — |
|  | Accumulation ratio | 17.9 ± 14.1 | — | — | — |

Accumulation ratio was defined as the ratio of AUC_0-8h_ on day 15/AUC_0-8h_ on day 1 in study NP25163, and as the ratio of AUC_0-8h_ on day 21/AUC_0-8h_ on day 1 in study YO28390.

*PK* pharmacokinetic, *SD* standard deviation, *CV* coefficient of variation, *AUC_0-8h_* area under the concentration-time curve from 0 to 8 hours, *C_max_* maximal plasma concentration, *C_trough_* plasma trough concentration, *t_½_* terminal half-life
