# Supplementary material for: Vemurafenib in Chinese patients with BRAFV600 mutation–positive unresectable or metastatic melanoma: an open-label, multicenter phase I study
Source: BMC Cancer. 2018 May 3;18:520. doi: 10.1186/s12885-018-4336-3 (PMC5934791; doi:10.1186/s12885-018-4336-3)
Supplement: Supplementary file 5 — Table S2. Comparison of efficacy between study YO28390 (Chinese patients) and the BRIM-2 and BRIM-3 studies (predominantly Caucasian patients). (DOCX 18 kb) [file 12885_2018_4336_MOESM5_ESM.docx]

**Table S2** Comparison of efficacy between Chinese and Caucasian patients

|  | Chinese patients  (study YO28390) | Caucasian patients | |
| --- | --- | --- | --- |
|  |  | BRIM-2 study [13] | BRIM-3 study [10] |
| Follow-up duration, months, median (range) | 11.3 (3.3–16.0) | 12.9 (0.6–20.1) | 12.5 (IQR, 7.7–16.0) |
| Confirmed BORR, %  (95% CI) | 52 (37–67) | 57 | 57 |
| Duration of confirmed response, months, median (95% CI) | 9.1 (7.4–NE) | 6.7 (5.6-8.6) | — |
| Median PFS, months  (95% CI) | 8.3 (5.7–10.9) | 6.8 (5.6–8.1) | 6.9 (6.1–7.0) |
| Median OS, months  (95% CI) | 13.5 (12.2–NE) | 15.9 (11.6–18.3) | 13.6 (12.0–15.2) |

*IQR* interquartile range, *BORR* best overall response rate, *CI* confidence interval, *NE* not estimable, *PFS* progression-free survival, *OS* overall survival
