# Supplementary material for: Vemurafenib in Chinese patients with BRAFV600 mutation–positive unresectable or metastatic melanoma: an open-label, multicenter phase I study
Source: BMC Cancer. 2018 May 3;18:520. doi: 10.1186/s12885-018-4336-3 (PMC5934791; doi:10.1186/s12885-018-4336-3)
Supplement: Supplementary file 6 — Table S3. Comparison of safety between study YO28390 (Chinese patients) and the BRIM-3 study (predominantly Caucasian patients). (DOCX 18 kb) [file 12885_2018_4336_MOESM6_ESM.docx]

**Table S3** Comparison of safety between Chinese and Caucasian patients

| AEs of any grade, n (%) | Chinese patients  (study YO28390) | Caucasian patients  (BRIM-3 study) [10] |
| --- | --- | --- |
| At least 1 AE | 46 (100) | 334 (99) |
| Dermatitis acneiform | 30 (65.2) | 17 (5) |
| Rash | 2 (4.3) | 138 (41) |
| Arthralgia | 30 (65.2) | 189 (56) |
| Blood cholesterol level increase | 27 (58.7) | 3 (<1) |
| Diarrhea | 27 (58.7) | 120 (36) |
| Blood bilirubin level increase | 25 (54.3) | 29 (9) |
| Melanocytic nevus | 24 (52.2) | 33 (10) |
| Alopecia | 23 (50.0) | 161 (48) |
| Palmar-plantar erythrodysesthesia syndrome | 22 (47.8) | 30 (9) |
| Photosensitivity reaction | 17 (37.0) | 137 (41) |
| Fatigue | 14 (30.4) | 156 (46) |
| Pyrexia | 13 (28.3) | 71 (21) |
| Rash maculopapular | 12 (26.1) | 34 (10) |
| γ-glutamyltransferase level increase | 11 (23.9) | 23 (7) |
| Proteinuria | 11 (23.9) | 2 (<1) |
| Total bile acid level increase | 10 (21.7) | 0 |
| Hypertriglyceridemia | 10 (21.7) | 2 (<1) |
| Leukopenia | 10 (21.7) | 0 |

*AE*, adverse event
